# Use of insecticide-treated clothes for personal protection against malaria: a community trial

**DOI:** 10.1186/1475-2875-5-63

**Published:** 2006-07-27

**Authors:** Elizabeth W Kimani, John M Vulule, Isabel W Kuria, Fredrick Mugisha

**Affiliations:** 1 African Population and Health Research Center (APHRC), P.O. Box 10787 00100, GPO, Nairobi, Kenya; 2 Kenya Medical Research Institute (KEMRI), PO Box 1578, Kisumu, Kenya; 3 Care International Kenya, P.O. Box 43864-00100 GPO, Nairobi Kenya

## Abstract

**Background:**

The study sought to determine the effect of using insecticide-treated clothes (ITCs) on personal protection against malaria infection. The specific objectives were to determine the effect of using ITCs on the rate of infection with malaria parasites and the effect on indoor mosquito density.

**Methods:**

This study was done in Dadaab refugee camps, North Eastern Province Kenya between April and August 2002, and involved a total of 198 participants, all refugees of Somali origin. The participants were selected through multi-stage cluster sampling. Half of the participants (treatment group) had their personal clothes worn on a daily basis (*Diras, Saris, Jalbaab*s, *Ma'awis *and shirts) and their bedding (sheets and blankets) treated with insecticide (permethrin). The other half (comparison group) had their clothes treated with placebo (plain water). Indoor mosquito density was determined from twelve households belonging to the participants; six in the treatment block and six in the comparison block.  During pre-test and post-test, laboratory analysis of blood samples was done, indoor mosquito density determined and questionnaires administered. Using STATA statistical package, tests for significant difference between the two groups were conducted.

**Results:**

Use of ITCs reduced both malaria infection rates and indoor mosquito density significantly. The odds of malaria infection in the intervention group were reduced by about 70 percent. The idea of using ITCs for malaria infection control was easily accepted among the refugees and they considered it beneficial. No side effects related to use of the ITCs were observed from the participants.

**Conclusion:**

The use of ITCs reduces malaria infection rate and has potential as an appropriate method of malaria control. It is recommended, therefore, that this strategy be considered for use among poor communities like slum dwellers and other underprivileged communities, such as street children and refugees, especially during an influx to malaria-prone regions. Further research on cost-effectiveness and sustainability of this strategy is worthwhile.

## Background

Malaria is the world's most prevalent vector borne disease. Each year 300 to 500 million clinical cases of malaria occur and at least 1 million people die of malaria each year [1]. About 90 percent of all malaria deaths in the world today occur in sub-Saharan Africa (WHO 2002). Since it increases morbidity and mortality, malaria slows economic growth in Africa by up to 1.3 percent each year [2].

The poor bear the highest burden of malaria: they are at a higher risk of becoming infected with malaria, because they live in dwellings that offer little protection from mosquitoes yet they may not afford protection methods like insecticide-treated nets (ITNs) [3]. Children from poorer households have higher mortality rates of which a substantial proportion is due to malaria. In Tanzania, a demographic surveillance system found that under-five mortality following acute fever (much of which would be expected to be due to malaria) was 39 percent higher in the poorest socioeconomic group than in the richest [4]. In a Zambian survey, the poorer exhibited higher malaria prevalence rates [5], while in Ghana, researchers found that the cost of malaria care was as much as 34 percent of the income of poor households, while it was only one percent of the income of the rich [6]. Malaria is also one of the most commonly reported causes of death among refugees and has caused high rates of both illness and death among refugees and displaced persons in endemic countries [7].

The WHO "Action Plan for the reduction of reliance on DDT in Disease Vector Control" recommends research into "the effectiveness, sustainability, and affordability" of insecticide-treated materials as one of the handful of approaches to replacing DDT use in malaria vector control [8]. ITNs have widely been tested in the control of malaria and have shown a great potential in reducing both morbidity and mortality due to malaria [9-14]. However, many people do not own nets and even less own ITNs. From demographic surveillance surveys, in nine countries surveyed between 1997 and 2001, a median 13 percent of households possess one or more nets, while a median 1.3 percent of households surveyed in three countries own at least one ITN [3].

According to Kenya Demographic Survey (KDHS) 2003, despite the fact that two third of Kenyans live in malaria-endemic areas, only 22 percent of households in Kenya have at least one mosquito net, while only six percent have at least one ITN. Only 10 percent have more than one net, while three percent have more than one ITN, despite the fact that an average Kenyan household size is 4.4 persons [15]. Studies have shown that various factors deter use of ITNs. Cost has been implicated as one of the major reasons for non-ownership of nets [16-18]. The populations most at risk are often among the poorest and may not always afford the cost of purchasing ITNs [19]. According to KDHS, 2003, the situation of net ownership in Kenya was reported to be worse for households in the lowest wealth quintile; only 11 percent have at least one net and worse still, only 2.5 percent have at least one ITN [15]. Cost is not the only factor that hinders ownership and use of nets; other important factors that hinder ownership and/or use of nets include size of houses, type and availability of sleeping facility and sleeping arrangements especially in refugee camps and other needy communities. Research has found that sleeping space determines whether it would be possible to hang a net. When the house is too small, it may not be feasible to use a net [20]. Houses in refugee camps and Daadab in particular are mainly makeshift houses and are small. Being located in a semi-arid area, temperatures in Dadaab are very high even at night. This sometimes makes the heat in the small house unbearable. Given the small size of the house, the number of people in one household (an average of six), and the heat in the house, some members of a household opt to sleep outside the house. Sleeping outside the house makes it rather difficult to use bed nets despite the fact that it puts the victims at high risk of mosquito bites. Like in many refugee camps and other poor communities, many of the households in the Dadaab refugee camps do not have beds and most of the few that have mostly have one bed which in many cases is improvised in that it is an elevation from the ground using earth. Therefore, most of the members who sleep inside the house sleep on the floor. Some studies have shown that if a person does not have a bed, the household puts more effort and higher priority on purchasing a bed than a bed net [21,22]. Another factor affecting use of nets is sleeping patterns; in most instances, only the parents use bed nets where they are available thus only the young children who sleep with the parents are protected while the older children are not protected [17].

These problems associated with use of ITNs limit their use especially in poor communities and other underprivileged communities like refugees. This points out to the need to utilize less expensive alternatives and more appropriate malaria control strategies in such communities. One potential method for malaria vector control that may meet the needs of these groups is insecticide-treated clothes (ITCs). However little research and consequently social marketing of the strategy has been done.

Results of a randomized controlled study done in Afghan refugee camp in 1996 showed potential of ITCs in reducing malaria infection for people aged less than 20 years [23]. The current study explored the effect of using ITCs on personal protection against malaria infection in a Kenyan refugee camp set-up. The study sought to determine whether use of ITCs would affect malaria infection rates and indoor mosquito density and whether the strategy would be acceptable to the community. In the Afghan study, malaria infection was detected through passive case detection at the camp's health centre. In situations where a low proportion of malaria morbidity is seen in formal health facilities for example in Africa [3], waiting for cases at the health centre could lead to a bias in reporting and consequently underestimation of the effect. Therefore, the current study used active case detection in the community to determine malaria infection rate as opposed to passive case detection at the health facility.

## Methodology

### Setting

The study was undertaken between April and August 2002 in Dadaab refugee camps, Garissa district, Kenya. In Dadaab refugee camps, mosquitoes breed mainly in pools formed by tap stand spillage. Other breeding sites included borrow pits, water pools formed during rains and animal watering cans.

There are three sub refugee camps in Dadaab; Ifo, Hagadera and Dagahaley. All the sub camps are divided into sections, which are further sub-divided into blocks. An average of nine blocks makes a section. Each block consists of about 600–700 people and consists of several households (which may consist of a nuclear family, an extended family or a group of unrelated refugees living under one roof) each consisting of an average of six people. The refugee population in the three camps is about 130,000 people; about 97 percent are from Somalia, while about three percent are Ethiopians, Sudanese, Ugandans and Eritreans. The study was undertaken in the Ifo sub-camp with a total population of about 45,000 people.

The Somali refugees are predominantly Muslim and wear long clothes; the women wear a veil or wrap known as *dira *(in some cases a light veil known as *galbasaar [sari] *is worn by young women) and a long dress known as *jalbaab *to cover the head and the rest of the body respectively. Similarly, the men wear a long skirt-like wrap known as *ma'awis*.

Many of the refugees have no source of income and rely almost entirely on assistance from aid agencies operating in the area including UNHCR, WFP, Care International, MSF-Belgium, and GTZ. They mainly live in makeshift houses some made up of earth and others of polythene paper, old clothes or sacks.

### Study design

This was a community trial which had a treatment arm and a comparison arm. Pre-test was done at the beginning of the study, then the intervention was given, the participants were followed up for about three months and post-test was done.

The study sample included refugees (males and females) of Somali origin of all ages who did not have any plans of moving from the camps for the six months following the initiation of the study and had no known severe skin disease, known allergic reactions to chemicals or severe respiratory problem.

Multistage cluster sampling was employed to select a sample of 198 participants; at the first stage, Ifo sub-camp was selected from the three sub-camps purposely because it was reported to have higher morbidity from malaria, at the second stage, two sections from Ifo sub-camp were selected at a distance of about one and a half kilometers apart (to cater for mosquito flight range). At the third stage, one block from each of these sections were selected randomly; one block was assigned treatment and the other comparison block randomly.

At the fourth stage, from the two blocks, 30 households (14 in the treatment block and 16 in the comparison block) were selected by systematic random sampling from a total of about 200 households; 100 in each block (therefore about 15 percent of the households in each block participated in the study). All the individuals residing in the selected household were recruited into the study. A total of 198 participants, 97 in the treatment and 101 in the comparison block were recruited.

Those in the treatment group had their clothes treated with insecticide while the comparison group had their materials treated with a placebo (plain water). The treatment of clothes was done by the lead researcher assisted by field assistants who were trained before the start of the intervention. The research participants were not allowed to handle the chemical and the chemical was also not being sold in the local market thus chances of cross-intervention were remote. In addition, treatment being done to personal clothes made any possibility of giving out treated materials to those in the comparison group remote, the effect of which would create some level of bias.

Double blinding was done for malaria parasite smear in that the participants did not know whether they were in the treatment or the comparison group and the laboratory technologist (who analysed the slides) was also not told which slides belonged to the treatment or comparison group.

### Execution

#### Pre-intervention

Training of field assistants was done in the first week of the study before the intervention. The field assistants were recruited from among Care Community Health Workers and MSF trained auxiliary nurses. After the training of field staff, with their assistance, community mobilization was done by first briefing the community leaders (*Gudomeyas*) on the study and then through their assistance, the community was mobilized. This was done in the second week before intervention was done.

#### Baseline survey

Baseline data was collected from participants in the households selected to participate in the study after community mobilization and before the intervention was given. The baseline data included presence of malaria parasites in blood samples taken from participants, indoor mosquito density, sleeping behaviours, malaria control practices and demographic data. Those found to have malaria parasites were referred to the refugee health post for treatment with the government recommended anti-malarial drugs (sulfadoxine-pyrimethamine). This was done to clear the participants of malaria parasites before the start of the study.

#### Intervention

Treatment of personal clothes (*diras, saris, jalbaab*s, *ma'awis*, and shirts) and beddings (sheets and blankets) with insecticide was done for the treatment group. Permethrin (Peripel EC 55) manufactured by AgrEvo at a concentration of 15 mls/4000 mls of water was used for treating the fabrics through dipping. The treatment was repeated every after three weeks during the study period. The treatment was repeated following the recommendations by the manufacturers of the insecticide that re-treatment should be done every after about five washes and the community indicated they washed personal clothes about twice a week. The comparison group had their materials dipped in plain water (acting as a placebo) at same intervals.

#### Post-intervention data collection

After the treatment of clothes, participants were followed for three months to measure the effect of ITCs on malaria parasite rates and on indoor mosquito density. Peripheral blood samples were taken twice after the intervention (about six weeks apart). The first post intervention test was done about two weeks after the treatment of clothes (which was about 3 weeks following the pre-intervention malaria parasite test). The samples were taken by field staff who had been trained as auxiliary nurses by MSF. The blood samples were stained with Giemsa and analysed using a light microscope. The laboratory analysis was done by a trained lab technologist from MSF at the MSF laboratory. The laboratory analyst was blinded on which slides belonged to the intervention or control group to reduce bias.

For those whose blood samples were found to have malaria parasites, they were taken to the MSF health facility and were treated with anti-malarial drugs (sulfadoxine-pyrimethamine). For blood samples, participants were tracked for three days after which they were regarded as absent. Thus, during the second and third post intervention period, some of the participants could not be traced. Most of those missing were reported to have gone on vacation to a different sub-camp.

Indoor mosquito density was also measured twice after intervention, about one week after blood samples were taken. Mosquitoes were collected from the houses at around six to seven in the morning using pyrethrum spray catch method. Each household was visited once per period. The collection of mosquitoes was done by field staff from vector and pest control unit in Care-Refugee assistance project (Care-RAP). Analysis of the mosquitoes was done with assistance of an entomologist from the vector and pest control unit, Care-RAP. Mouthparts were used to identify the species and sex of the mosquitoes. The status of the mosquito, whether engorged or not was established. Identification was done by help of a dissecting microscope. Participants were observed during the follow-up period for side effects including reactions to the skin, effect on the respiratory system or any complaints/symptoms. A simple survey was done at the end of the study through interviewing households to determine the experiences in using the treated materials and rates of self reported malaria.

### Data analysis

Using the STATA statistical package, analysis involving comparing the two samples (treatment and comparison) was done. Odds Ratio (OR) was calculated to determine difference between the treatment and the comparison group. Poisson regression was done for mosquito density while logistic regression analysis was done for malaria infection status. For the logistic regression, various factors that could be associated with malaria infection were controlled for in the regression models. Cases with at least one record of information on the dependent variable (malaria infection status) during the follow-up period after intervention were considered. If there was no data collected on cases regarding their malaria infection status during the two data collection periods following intervention, the cases were deleted (seventeen cases in total). For all the tests statistical significance was assumed at 5 percent level.

### Ethical considerations

The study was approved by the Moi University institutional ethical clearance board before it was undertaken. The fundamental principles of ethics in research on human participants were upheld throughout the project. The research procedures were disclosed to the participants and informed consent was sought from the participants through a signed consent form. Nobody was coerced into the study and if one wished to withdraw, they were allowed to do so without prejudice; two households actually withdrew from the study. This study was supported by Care International Refugee Assistance Project and there was plan that should the strategy be found to be effective, Care would implement the strategy in the refugee camps thus the comparison group would have their clothes treated with the insecticide.

## Results

### Pre-intervention period

#### Characteristics of the study participants and households

A total of 198 participants from 30 households were admitted into the study; 97 participants in the treatment group from 14 households and 101 in the comparison group from 16 households. Blood samples for malaria parasite smear were taken from 193 participants; 96 from treatment group and 97 from the comparison group. Mosquito density was assessed from 6 households in each group. As seen from Table 1, there was no statistically significant difference between the two groups of participants as regards their individual characteristics including sex, age, whether they slept outside the house or not and their malaria infection status. However, there was a statistically significant difference in the proportion of households with mosquito nets and in mosquito densities; the control group had significantly more households with mosquito nets and the density of indoor mosquitoes was significantly less. The two groups being in the same sub-camp were from the same geographical area.

**Table 1:** Characteristics of Study Participants at Admission

	**Treatment Group**	**Comparison Group**
**Individual Characteristics**		
Number recruited	97	101
Sex (male percent)	57	51
Age (Mean years)	16.65	18.60
Sleeping outside (percent)	42	38
Malaria Infection Status (percent positive)	13	8
**Household Characteristics**		
Number of Households	14	16
Possession of Mosquito Nets (percent)	24	67*
Total Mosquito density (mean)	34	9.33*
Engorged mosquito density	10.67	2.17*
Female mosquito density	18.83	5.17*
Anopheles mosquito density	7.67	1.17*
Female Anopheles mosquito density	2.67	0.17*

*Difference between treatment group and comparison group significant at P = 0.05

### Post-intervention period

#### Indoor mosquito densities

During the post-intervention period, the number of mosquitoes collected from the treatment block decreased compared to the number collected during pre-intervention period. In contrast, the number of mosquitoes collected from the comparison group increased compared to the number collected during pre-intervention. Reduction of mosquito density in the intervention households was significant for total mosquito density, engorged mosquitoes (mosquitoes that had fed on blood meal), female mosquitoes and anopheles mosquitoes but was not significant for female anopheles mosquitoes (Table 2).

**Table 2:** Indoor mosquito densities

Mosquito* Category		**Treatment Group**			**Comparison Group**		
	
	Parameter	Estimate	Std. Error	P-Value	Estimate	Std. Error	P-value
Total Mosquitoes	Intercept	3.526	0.070	<0.0001	2.234	0.134	<0.0001
	Pre-intervention	0.000	0.000		0.000	0.000	
	First post – intervention	-1.468	0.162	<0.0001	0.931	0.158	<0.0001
	Second post – intervention	-1.468	0.162	<0.0001	0.344	0.175	0.0489
Engorged	Intercept	2.367	0.125	<0.0001	0.773	0.277	0.0053
	Pre-intervention	0.000	0.000		0.000	0.000	
	First post – intervention	-0.981	0.240	<0.0001	1.019	0.324	0.0016
	Second post – intervention	-1.326	0.273	<0.0001	0.693	0.340	0.0413
Females	Intercept	2.936	0.094	<0.0001	1.642	0.180	<0.0001
	Treatment group	0.000	0.000		0.000	0.000	
	First post – intervention	-1.039	0.184	<0.0001	0.829	0.215	0.0001
	Second post – intervention	-1.262	0.200	<0.0001	0.373	0.233	0.1103
Anopheles	Intercept	0.037	0.147	<0.0001	0.154	0.378	0.6834
	Treatment group	0.000	0.000		0.000	0.000	
	First post – intervention	-3.829	0.011	0.0002	1.551	0.416	0.0002
	Second post – intervention	-1.750	0.383	<0.0001	0.134	0.518	0.7964

*Female anopheles mosquitoes were few and the change in density was not significant so they were omitted in this table

#### Malaria infection rates

During the post-intervention period, malaria parasite test was done twice. The status was indicated as positive if during any of the tests the participant had a smear-positive slide and negative if the two tests were negative for the individual (Table 3). Blood samples were collected from a total of 181 participants; 90 from the treatment group and 91 from the comparison group. As indicated in Table 4, malaria infection rate was higher in the comparison block than in the treatment block. The proportion of those who got infected with malaria after intervention in the comparison block was 66 percent while in the treatment block the proportion was 38 percent. The Odds Ratio was reduced by 69 percent (OR = 0.31, P < 0.001) for the treatment group. When the analysis for malaria infection was done for different age groups, results showed that the intervention was protective for children aged 5–14 years, youth aged 15–24 years and for those aged over 50 years but was not protective for adults aged 25–49 years and children less than five years.

**Table 3:** Odds of malaria infection post-intervention

**Age category**	**Odds ratio**	**P-value**
All Ages	0.314	0.0002
Under five (0–4 years)	0.564	0.3489
Children (5–14 years)	0.237	0.0086
Youth (15–24 years)	0.086	0.0113
Adults (25–49 years)	0.714	0.5913
Older people (50+ years)	0.057	0.0201

**Table 4:** Predicted logistic regression Odds Ratio of being infected with malaria parasites

	**Model 1**	**Model 2**	**Model 3**
Intervention status (Treatment)	0.314*	0.301*	0.286*
Possess nets(Yes)		0.909	0.860
Use other methods to control malaria (Yes)			0.982
Sleep outside (Yes)			1.271
Sex (Female)			0.732
Age category (under five years)			
5–14y			0.925
15–24y			1.263
25–49y			0.984
50+y			0.517
Df	1	2	9
-2loglikelihood	236.082*	236.006	233.194

*Significant at P = 0.05

A logistic regression was done with malaria infection status as the dependent variable to control for various factors that could affect malaria infection rate (Table 4). Clustering at the household level was controlled. In the first model, regression of overall malaria status against intervention status (whether one was in the intervention group or not) was done. The odds of being infected with malaria if one was from the treatment group were 0.31 (P = 0.001). Since possession of nets was found to be significantly different in the two blocks, possession of nets was added in the model. Therefore, in the second model, the possession of nets was controlled for to see if the effect changes. The Odds Ratio changed to 0.30 (P = 0.002), thus the odds of being infected with malaria was reduced by 70 percent if possession of nets was controlled indicating that since the comparison block had a higher possession of nets, the nets offered them some protection, therefore, reducing the overall calculated effect slightly when possession of nets was not controlled.

In the third model, several characteristics including possession of nets, sex, age, use of other malaria control methods and sleeping outside were added to the model. These factors may not be directly related to malaria infection but they can alter the risk of infection with malaria parasites. When all these factors were controlled, the odds Ratio changed to 0.29 (P < 0.001). This indicates that the odds of being infected with malaria when all these factors were controlled were reduced by 71 percent.

### Experiences with the intervention

#### Acceptability

At the on-set of the study, the community was very enthusiastic about the study and everyone selected accepted to be part of the study. However, during the last post-intervention data collection, one household from the comparison group withdrew completely from the study saying the study had no benefits to them. This household refused to have blood samples taken from the members during the last post-intervention period and also to have mosquitoes collected from their household in the last post intervention data collection period. This household was replaced with the immediate neighbouring house for mosquito density, but was not replaced for malaria infection status. Another household from the comparison group refused to have blood samples taken from them during the last post intervention period but allowed mosquitoes to be collected.

#### Perceptions of morbidity from malaria

The information on perceptions of malaria morbidity was collected during the last month of the study. Participants were asked whether any member of the family had suffered from malaria during the past one month. About nine percent of the participants were reported to have had malaria during the past one month. More people in the comparison group (13 percent) were reported to have had malaria than in the treatment group (5 percent) and the difference was statistically significant (OR= 0.33; P = 0.05).

#### Harm versus benefits from the treatment

Observation for side effects was done during the follow-up period. No side effects were observed among the participants or reported by the participants from the use of the treatment throughout the study period. All the participants in the treatment group said the treatment was advantageous. Among the advantages given were that use of the treated clothes reduced the mosquitoes in the house and mosquito bites, and that other insects like bedbugs and body/head lice in children were reduced. They also said that children no longer had problems with sleep at night because there was no nuisance from mosquito bites; that children hardly woke-up in the middle of the night and slept until 8 am in the morning nonstop, as opposed to earlier times when they used to wake up at night crying.

Those in the comparison group said they perceived no benefit from the study apart from two households who said mosquitoes were reduced but only for the first one night after intervention. One family from the comparison group reported that they thought that the treatment was attracting mosquitoes to their household. This is the household that withdrew completely from the study.

## Discussion

This study shows that insecticide-treated clothes are protective against malaria. The odds of being infected with malaria for those in the treatment group were significantly reduced when all ages were combined. When the odds were calculated by age groups, the odds were significantly reduced for older children (age 5–14years), youth (age 15–24years) and the older people (50 years and above). For children less than five years and the adults 25–49 yeara, the results showed no benefit. This study adds to the body of knowledge on the potential for ITCs or ITMs as an alternative to ITNs. A similar study of relevance is a trial of permethrin-impregnated bedsheets (*shukas*) in the pastoral community in Kenya [24]. In their study, they enrolled a total of 472 individuals in a randomized community trial where the unit of randomization was the hamlet (manyatta). They dipped bedsheets owned by the experimental group in permethrin. They found that the prevalence of malaria in the study population (based on laboratory results) was considerably lower than that used for the power calculation based on clinical estimates.

The results found in this study also support what Rowland found in his study on insecticide treated *chaddars *and top sheets amongst Afghan refugees [23], where the odds of having malaria episode were significantly reduced in refugees aged less than 20 years in the group using permethrin-treated *chaddars *and top-sheets. The results are also comparable to results found while using ITNs [9,10,13,14]. However, nets have been found to have various shortcomings and their usage is thus compromised [16-22]. ITNs are expensive and often impractical for some communities for example refuges, pastoral communities, communities in hot climates and the poor. Despite the fact that use of nets deter mosquitoes from entering the houses and reduces mosquito bites, research has shown that some people who report having nets in the households do not use them, for example, in the 1992 Malawi National Survey, only about 66 percent of households with nets reported that they had been used the previous night [16]. Even when people report that they use the nets, studies that have included night visits have shown that the reported use is usually higher than the actual use. In one efficacy trial in western Kenya, 85 percent of people with nets claimed to use them regularly, but night visits indicated a use rate of about 70–73 percent during the dry season. Non-users said either that they had "forgotten" or that it was too hot for nets [25]. Possible non-compliance to use of nets for those who possess them may explain why the results showed that possession of nets did not significantly affect malaria status in the current study. Other studies have shown that net owners still sit outdoors when mosquitoes are biting and sleep outdoors in hot weather or before the harvest, when people sleep in the fields to protect crops from animals [26]. This could be a further explanation why nets in this community did not significantly protect the owners since a large proportion of the study participants were reported as sometimes sleeping outside the house especially when it was too hot in the house while many young men in this community sat outside till wee hours of the night chewing "miraa" (khat).

In the current study, despite the fact that the region where Dadaab refugee camps are located has low risk of malaria generally, the risk of malaria infection at the camps may be higher than in the surrounding areas because of poor sanitary practices at the camps. The refugees in Dadaab fetch water from communal taps located at several positions in the camps. Water spilled at the tap-stands contributes a lot in breeding of mosquitoes in the refugee camps. The overall percentage of those infected with malaria parasites before the intervention was low compared to the months after the intervention. This could be explained by the season preceding the start of the study, which was quite dry. By the time the first post-intervention data was collected, the rains had somehow stopped but there were water pools still lying about. During this period, the indoor mosquito densities in the comparison group increased as compared to the pre-intervention densities. This could be due to weather changes: mosquitoes bred in the water pools formed by the rains because though the rains had stopped, the flat terrain and poor drainage of the area encouraged persistence of the water pools and, thus, mosquito breeding. If nature was left to take its own course, the same would have been expected to happen in the comparison group. However, the densities in the treatment group decreased as compared to the pre-intervention period. This could be explained by the intervention given to this group because as expected with permethrin, most mosquitoes coming to the house were either repelled by the ITCs or were knocked down. Accordingly, there were fewer blood-fed mosquitoes in the treatment group as compared to the comparison group (though difference not significant). For the comparison group, this could be explained by the fact that mosquito breeding had increased following the rains. During the second post-intervention data collection, the rains had stopped and the water pools formed by the rains had somehow dried up. Most breeding of mosquitoes was happening at the tap-stand spillage and at pit latrines (for *Culicine *mosquitoes). This explains why there were very many *culicine *mosquitoes and very few *Anopheles *mosquitoes among the mosquitoes collected in the houses. The density of mosquitoes collected from the treatment block was less than that of the comparison block.

The cost of insecticide-treated materials may be much lower than the cost of ITNs. In the current study, the cost benefit analysis were not done but in the Afghan study, the cost of permethrin treatment per person protected (US$ 0.17) was similar to that of treating bed nets and cost only 10–20 percent of the price of a new bed net [23]. This points out to the affordability of the strategy to the vulnerable groups and with the fact that this strategy does not require people to change their sleeping patterns like bed nets do, the possibility of its sustainability may be high. This indicates that where nets are unavailable the importance of this strategy cannot be overemphasized and where nets are not adequate, this strategy could be employed as a complement to the use of nets. Despite the potential of using insecticide treated clothes in malaria transmission especially in vulnerable communities, they have not been marketed as much as the more expensive and less appropriate mosquito nets in the vulnerable communities. Social marketing has mainly been targeted to mosquito nets and many governments in the developing countries, Kenya being a good example have shown commitment of promoting net use. For example in Kenya, according to the national malaria control strategy, the goal is to have 60 percent of households use nets by 2006. Unfortunately, such targets are usually to households and not to individuals due to the cost of providing the nets.

Notwithstanding the findings on the potentiality of ITCs in malaria infection control in this study, further research requires to be done to assess cost-effectiveness and sustainability of this strategy.

## Conclusion

It was concluded that use of ITCs reduces mosquito densities in the house and the rate of infection with malaria parasites. The reduction in the nuisance caused by mosquito bites cannot be overemphasized. It is recommended that this strategy be considered for use in the control of malaria and nuisance caused by mosquito bites in vulnerable communities like poor communities living in slum areas as poor people are less likely to afford ITNs, and if they do, they may not afford enough ITNS to cater for the whole household and in such a circumstance, ITCs could complement the ITNs. The strategy is also recommended for refugee communities especially during emergencies like during influx of refugees to a malaria-prone region as some refugees may not have immunity to malaria. However, a larger scale study for a longer period to traverse different seasons and to determine sustainability and possibly cost-effectiveness of the strategy in a community set up is worthwhile.

## Authors' contributions

KEW conceived and designed the study, collected and analysed the data, drafted the manuscript and gave final approval for publishing. VJM contributed to the design of the study, supervised data collection, reviewed the manuscript and gave final approval for publishing. KIW contributed to the design of the study, participated in data collection, reviewed the manuscript and gave final approval for publishing. MF contributed to the analysis of data, reviewed the manuscript and gave final approval for publishing.
